# Outcomes after Hysteroscopic Treatment of Symptomatic Isthmoceles in Patients with Abnormal Uterine Bleeding and Pelvic Pain: A Prospective Case Series

**DOI:** 10.22074/ijfs.2019.5704

**Published:** 2019-04-27

**Authors:** Ana Vegas Carrillo de Albornoz, Irene López Carrasco, Nerea Montero Pastor, Carmen Martín Blanco, María Miró Matos, Luis Alonso Pacheco, Enrique Moratalla Bartolomé

**Affiliations:** 1 Department of Obstetrics and Gynecology, University Hospital HM Montepríncipe, Madrid, Spain; 2 Department of Gynaecological Endoscopy, Gutenberg Center, Xanit International Hospital, Malaga, Spain; 3 Department of Obstetrics and Gynecology, University Hospital Ramón y Cajal, Madrid, Spain

**Keywords:** Caesarean Section, Hysteroscopy, Infertility, Metrorrhagia, Pelvic Pain

## Abstract

**Background:**

Isthmoceles are described as complications associated with caesarean section (CS). Only symptomatic isthmoceles should be treated. The main symptoms are abnormal uterine bleeding (AUB) in the absence of any other causes, pelvic pain and secondary infertility. There are several techniques described for the correction of isthmoceles. Isthmoplasty can be performed by hysteroscopy, laparoscopy or vaginal surgery. The aim of this study was to assess the effectiveness of hysteroscopic surgical treatment of isthmoceles in women with associated symptoms such as pelvic pain and AUB.

**Materials and Methods:**

A prospective case series study was performed; this study included all women with AUB, pelvic pain and ultrasonographic (US) diagnosis of isthmocele, who had undergone hysteroscopic correction between June 2014 and December 2017 in our Hospital.

**Results:**

Thirty eight women underwent surgical hysteroscopy for correction of symptomatic isthmoceles. All patients presented AUB, 42.1% experienced pelvic pain and 28.9% had secondary infertility. US evaluation of isthmoceles was performed using 2D ultrasound. The residual myometrial thickness (RMT) above the isthmocele was measured in women who expected future pregnancy; if it was <2.5 mm the patient was not included in the study because the cor- rection was performed laparoscopically. Follow-up was performed one and two months after the surgery. In all cases, pelvic pain was resolved one month after the surgery. AUB disappeared within the first month in 87.5% of patients and in the second month in 96.8% of subjects; however, one patient needed further surgery to alleviate her symptoms. Secondary infertility was assessed one year after surgical isthmoplasty. Seven women completed the first year of fol- low up, and three of them (42.8%) reported pregnancy after treatment between six and eight months after the surgery.

**Conclusion:**

Hysteroscopic correction of symptomatic isthmoceles may constitute a safe and effective technique for patients who present AUB and pelvic pain.

## Introduction

The number of deliveries by caesarean section (CS) has increased during the last 15 years (1). In 2016, the global rate of CS in Spain was about 22%, even higher in private hospitals (2). This rise probably leads to a greater incidence of complications. Uterine scars defects, also known as isthmoceles, are described as complications associated with CS. An isthmocele is an anatomical uterine defect, defined as a reservoir-like pouch in the isthmus of the anterior uterine wall, at the site of the CS scar (3-5). This complication is more frequently observed in women with retroverted uterus and those with multiple CS (6).

It is thought that the scar defect appears due to tissue healing impairment, probably secondary to reduced vascular perfusion in this area (6, 7). Although the mechanisms are unknown, several factors such as differences in the thickness between the superior and inferior edges of the hysterotomy (8), the stage of labour at the moment of the CS (9, 10) or the suturing technique (8, 11-14) may contribute to the formation of the defect. Isthmoceles are usually asymptomatic, and may be incidentally diagnosed by ultrasonography; in such cases, treatment is not required. The niche (anechoic area) may vary in sizes and symptoms may be related to the size of the defect (6, 15, 16). The main symptoms are abnormal uterine bleeding (AUB) in the absence of any other causes, pelvic pain and secondary infertility (3, 6, 8, 9, 17). The typical pattern of bleeding is postmenstrual dark spotting.

The presence of an isthmocele may also cause complications during some gynaecological procedures such as curettages, hysteroscopy, intrauterine device insertion or in embryo transfers, because of alteration of uterine anatomy (18). Diagnosis is based on the symptoms and complementary exams. Transvaginal ultrasound (TVUS) and hysterosonography measure not only symptomatic defects but also isthmoceles in asymptomatic patients (14, 16, 18-20). Hysteroscopy is also a very effective technique that ensures diagnostic confirmation by direct visualization of the pouch enabling direct correction of the defect (21).

The aim of this study was to assess the effectiveness of hysteroscopic surgical treatment in patients with pelvic pain, AUB and TVUS diagnosis of isthmocele, in the absence of other causes.

## Materials and Methods

This prospective case series study included all women with AUB, pelvic pain and US diagnosis of isthmocele, in the absence of other causes, who had undergone hysteroscopic correction between June 2014 and December 2017 in our hospital. The study was approved by the Ethical Committee of the Hospital with code 18.07.1270-GHM.

**Fig 1:**
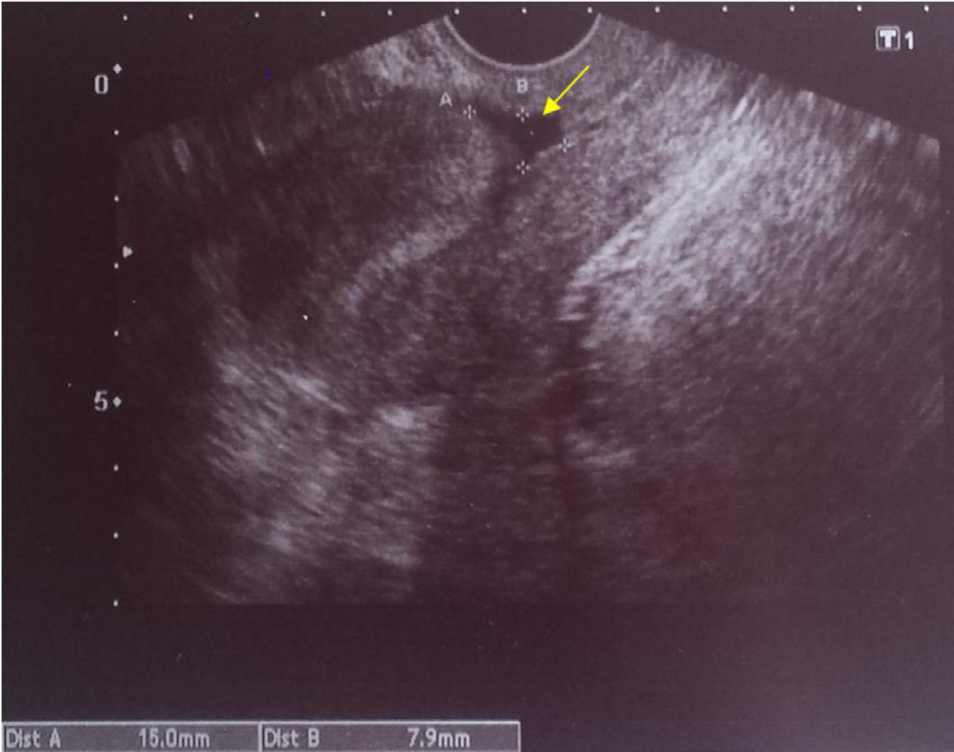
Ultrasound image of an isthmocele. Yellow arrow points towards the isthmocele.

The diagnosis was based on the symptoms, patients’ background once other possible causes of AUB had been excluded. As complementary test at office we used TVUS. TVUS was performed in early proliferative phase. The isthmocele is identified as an anechoic triangular-shaped area, with the vertex pointing to the bladder, in the isthmus of the anterior uterine wall. Both depth and width of the defect were measured (Fig .1).

Scar defects were classified based on their size according to the classification proposed by Gubbini et al. (4) using the triangle area formula: base x height/2. Gubbini et al. (5) established 3 grades as follows: grade I: <15 mm^2^; grade II: 16-25 mm^2^; and grade III: >25 mm^2^ (4, 5). The residual myometrial thickness (RMT) above the vertex of the isthmocele, was also measured in patients who expected future pregnancy. When the RMT was <2.5 mm, the correction of the defect was performed by laparoscopic technique and these patients were not included in this case series. All women were assessed by the anaesthesiology team and provided with informed consent.

**Fig 2:**
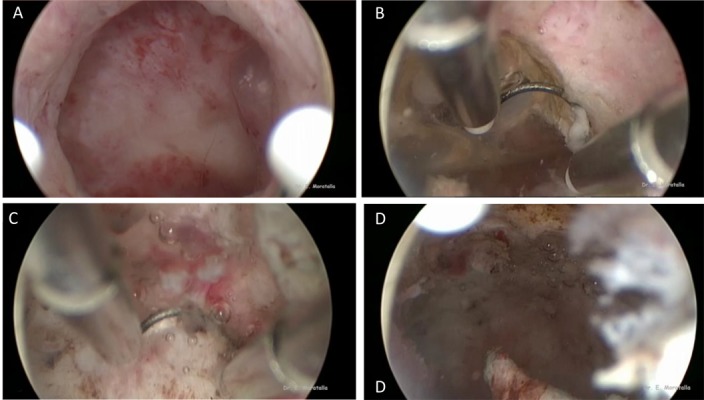
Hysteroscopic isthmoplasty. A. Isthmocele, B. Resection of the anterior arch, C. Coagulation of the bottom of the niche, and D. Image after resection.

Hysteroscopy was performed under general anaesthesia in the operating room, using saline solution as distending media. All hysteroscopies were done by two experienced surgeons who followed the same protocol. Initially, a diagnostic hysteroscopy using 5-mm, 30º angle lens, rigid hysteroscope (Karl Storz GmbH and Co, Tuttlingen, Germany), without cervical dilatation, was done in order to achieve direct view of the scar defect and to exclude other intrauterine anomalies. Afterwards, hysteroscopic niche resection was performed using a 9-mm bipolar loop resectoscope (Ethicon Gynecare Inc., Johnson and Johnson). Small defects were resected by a 5-mm hysteroscope and a 5-Fr bipolar electrode. Anterior and posterior fibrotic arch of the isthmocele were identified. The anterior arch was resected by the bipolar loop resectoscope or the 5-Fr bipolar electrode in cases of small defects, until the bottom of the isthmocele reached the level of the cervical canal. The bottom of the sacculation was coagulated (Fig .2).

## Results

Between June 2014 and December 2017, 38 patients underwent surgical hysteroscopy for correction of symptomatic isthmoceles. Mean age of the patients at the intervention was 40 [31-47] years. All women presented postmenstrual AUB (PAUB). Among them, 16 patients (42%) had pelvic pain and 11 (29%) had secondary infertility. All women had at least one previous CS (63.1%), nine women (23.6%) had 2 CS, and five women (13.1%) had 3 previous CS. Regarding the anatomical position of the uterus, 65.7 and 34.3% of patients presented anteverted and retroverted uterus, respectively. Isthmoceles were classified according the US based classification described above. Nine out of the 38 women (23.6%) presented grade 1 isthmocele, eight women (21%) presented grade 2 defects and 21 women (55.2%) had grade 3.

In 81% of cases (31 patients) the procedure was performed using a bipolar loop resectoscope as the diagnosis had already been established on a previous diagnostic hysteroscopy. For the rest of the patients (19%) who had smaller defects, correction of the isthmocele was carried out using a 5-Fr bipolar electrode. All patients were discharged on the day of the surgery. No complications or adverse effects were reported after hysteroscopic resection. The criterion for selecting a specific hysteroscopic resection technique was the size of the niche. The RMT was also taken into consideration in patients with secondary infertility who expected future pregnancy, those women who presented an RMT <2.5 mm were excluded from hysteroscopic correction and underwent laparoscopic correction of the isthmocele. Follow-up was performed 1 and 2 months after the surgery. PAUB was the most frequently reported complaint, which was resolved within 2 months in almost all women; however, 1 woman needed a second surgery to eliminate the spotting. In 79.5% of patients, PAUB disappeared within the first month, and after two months of follow-up, 97.4% of women did not present with AUB. Pelvic pain was resolved in 100% of the patients 1 month after surgery.

Ultrasonographic (US) follow-up showed that after the surgery, 100% of grade I and II isthmoceles were completely resected. On the other hand, in three of the twenty one grade III isthmoceles, despite the resolution of the symptoms, small defects could still be observed on US, two months after the surgery (Table 1).

**Table 1:** Results of ultrasonographic follow up


Ultrasonographic image	Before surgery	1 month after surgery	2 monthsafter surgery

Grade I	23.6%, n=9	0	0
Grade II	21%, n=8	0	0
Grade III	55.2%, n=21	4 grade I	3 grade I


**Fig 3:**
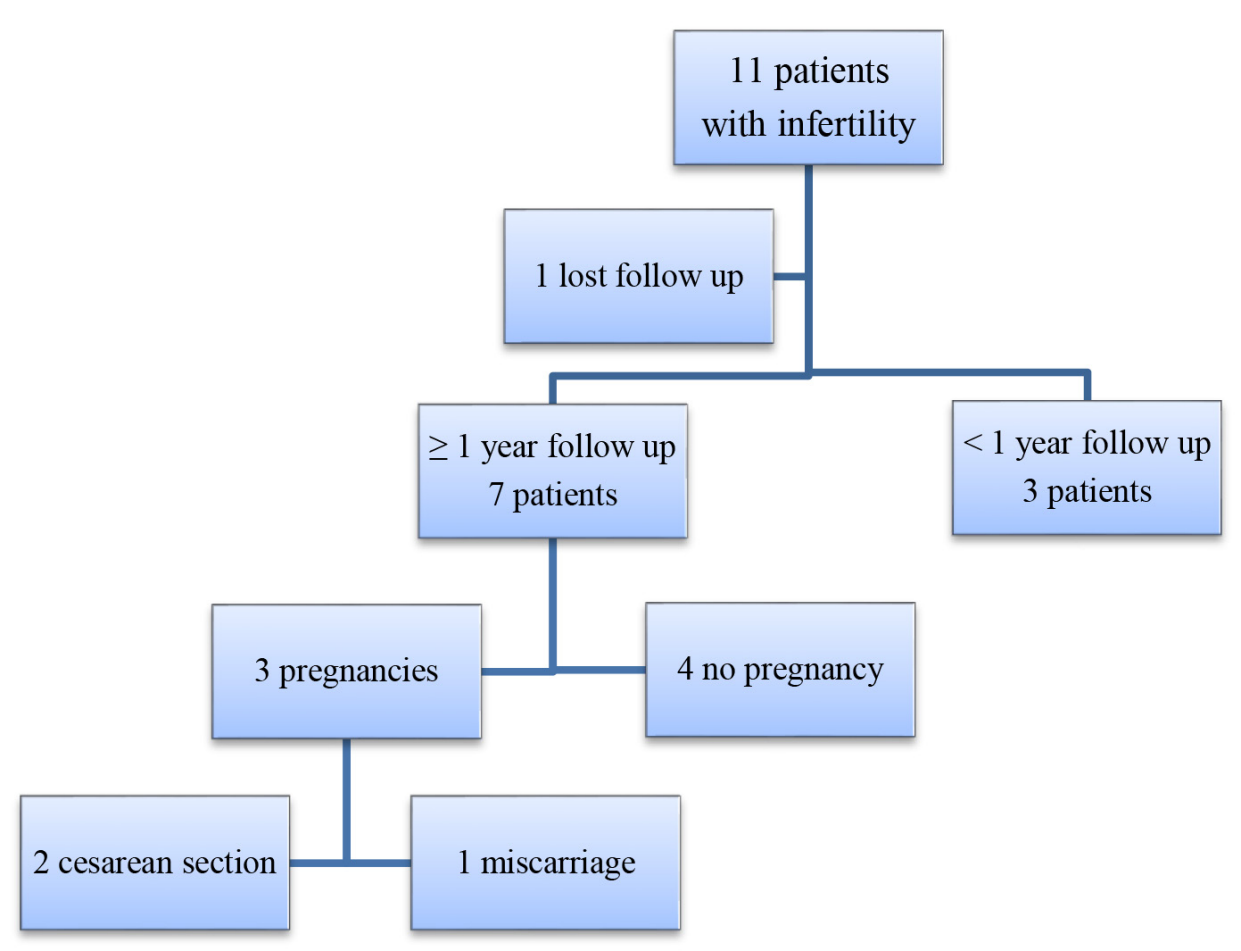
Follow up and results in patients with secondary infertility after hysteroscopic isthmoplasty.

Secondary infertility was assessed one year after surgical isthmoplasty. Eleven patients showed infertility, seven completed the first year of follow up, and three of them reported pregnancy after treatment (42.8%) between six and eight months after the surgery. One patient was lost to follow-up, and the remaining three women have not yet completed one year of follow-up. Among the patients who reported pregnancy, one presented a miscarriage after 7 weeks of pregnancy and in the two other cases, pregnancy evolved without incidents undergoing CS after 38 weeks of pregnancy (Fig .3).

## Discussion

Postmenstrual AUB (PAUB) is the most frequent complaint among patients with symptomatic isthmoceles. In 1995, Morris (3) was the first to describe the caesarean scar defect. He examined the uterus of women who had undergone hysterectomy due to AUB symptoms in the absence of any identifiable cause and did not respond to hormonal therapy. All women had at least one previous CS. He found that most of these women presented distortion and widening of the lower uterine segment as well as inflammatory changes in this site. It was proposed that menstrual blood accumulates in the isthmocele and delay menstrual bleeding, causing PAUB (18). Not only the anatomical defect is responsible for the spotting, but also other mechanisms such as, in situ production of blood (3) and decreased contractility of the myometrium in this area (22) were suggested to contribute to blood accumulation.

Surgical hysteroscopy enables correction of the anatomical defect by removing the edges of the niche, avoiding, in this way, the accumulation of the menstrual blood. In addition, the cauterization of the pouch of the isthmocele reduces the in situ production of blood and release of inflammatory factors, and produces a scar retraction of the pouch. Several authors, in non-controlled reports, suggested that these hysteroscopic procedures seem to be effective in improving isthmocele symptoms, even achieving the resolution of the AUB in the majority of the patients (3, 5, 14, 23-26). So far, only one controlled study was conducted to compare the resectoscopic treatment of symptomatic isthmoceles to the expectant management, reporting the complete resolution of symptoms in 87% of the treated patients, with a significant difference compared to untreated women (27). So, as we can see, our results are consistent with previous studies. In relation to secondary infertility, it is thought that the isthmocele produces a toxic environment due to the accumulation of blood and the release of inflammatory factors, obstructing the passage of sperms and preventing embryo implantation (5, 8, 17). Hysteroscopic correction of the isthmocele may also improve pregnancy outcomes (5, 23, 26).

Although no complications were reported after hysteroscopic isthmoplasty, it is important to consider that the surgical technique is not exempt from complications. Besides the general risks of hysteroscopy, in this case, it should be noted that the myometrium above the isthmocele is thinned, which implies a greater risk of perforation and therefore, vascular, bladder or bowel injury (28).

To prevent the risk of uterine perforation and bladder injury, it is recommended to measure the RMT above the isthmocele. In our series, patients with an RMT <2.5 mm were not included, because in such cases, the correction was performed laparoscopically, as suggested by Tanimura et al. (17). At the moment we began our study, there was controversy over the value of the RMT that was safe and recommended for the hysteroscopic correction of the isthmocele. They established the cut-off point of 2.5 mm for RMT, and Marotta et al. (28) and Donnez et al. (29) proposed the laparoscopic correction of isthmocele when the RMT above the isthmocele is <3 mm; on the other hand, Raimondo et al. (24) suggested to avoid hysteroscopic correction in patients with an RMT <4 mm. In 2018, the Global Congress on Hysteroscopy Scientific Committee (30) published a consensus statement for the management of symptomatic isthmoceles, establishing that when myometrial thickness is <3 mm, the laparoscopic approach is preferred to reduce the risk of perforation. This is the limit (cut-off) we are currently using. Moreover, in patients with secondary infertility, who expect future pregnancy and undergo isthmoplasty, it seems especially important to avoid excessive myometrial resection. In these cases, the goal will be to achieve a pregnancy, and extremely thin residual myometrium increases the risk of uterine rupture (17). Therefore, for patients who are looking for pregnancy and have a RMT <3 mm, laparoscopic correction is the recommended option, since it also favours the restoration of the myometrial thickness (17, 28, 29, 31).

Being conscious of the limitations of our study, a case series study with a limited number of patients, and knowing that more randomized control trial are needed to demonstrate the efficacy of the hysteroscopic treatment of symptomatic isthmoceles, it seems that this technique can be effective to resolve PAUB and pelvic pain in women with symptomatic isthmoceles. Another limitation of our study was the assessment of the fertility outcome as one year follow-up of patients who presented secondary infertility was difficult and some of them were lost follow-up.

## Conclusion

Isthmoceles constitute a frequent cause of AUB and pelvic pain in patients with CS. Therefore, isthmoceles should be included in the differential diagnosis of AUB and pelvic pain in premenopausal women with history of previous CS. Symptomatic isthmoceles should be treated. In patients with AUB or pelvic pain who do not expect future pregnancy, hysteroscopic correction of the isthmocele may constitute the first choice of treatment being a minimally invasive technique that improves the symptoms. On the other hand, in women who expect future pregnancy, it seems to be important to consider the RMT above the vertex of the isthmocele to select the best surgical technique for correction of the defect. Hysteroscopic isthmoplasty also seems to be a safe and effective technique in patients who present an RMT of >3 mm. Nevertheless, further studies are needed to determine the surgical technique and type of treatment which would be better for each patient.
